# Potential therapeutic targets for hypoxia-induced pulmonary artery hypertension

**DOI:** 10.1186/1479-5876-12-39

**Published:** 2014-02-08

**Authors:** Li Dong, Yuping Li, HongLing Hu, Lin Shi, Junjie Chen, Beibei Wang, Chaolei Chen, Haiping Zhu, Yunlei Li, Qiu Li, Liping Zhang, Chengshui Chen

**Affiliations:** 1 Department of Respiratory Medicine, The First Affiliated Hospital, Wenzhou Medical College, Wenzhou 325000, China; 2 Department of Intensive Care Unit, Medicine, The First Affiliated Hospital, Wenzhou Medical College, Wenzhou 325000, China; 3 Department of Respiratory Medicine, Yueqing people’s Hospital, Wenzhou 325000, China; 4 Department of Respiratory Medicine, Zhuji people’s Hospital, Shaoxin 312000, China; 5 Department of Intensive Care Unit, Medicine, Lihuili Hospital, Ningbo, 315000, China

**Keywords:** 5-hydroxydecanoate, Mitochondrial ATP-sensitive potassium channel, Hypoxia, Kv1.5 channel, Pulmonary artery hypertension

## Abstract

**Background:**

Hypoxic pulmonary artery hypertension (PAH) as a severe pulmonary disease is characterized by changes of pulmonary vascular reconstruction. Mitochondrial ATP-sensitive potassium channel (mitoK_ATP_) was considered as one of factors responsible for the proliferation of hypoxic pulmonary arterial smooth muscle cells (PASMCs), although the exact mechanisms remain unclear.

**Methods:**

Pulmonary artery hypertension was induced in rats with or without 5-hydroxydecanoate (5-HD). The mean pulmonary artery pressure, morphologic changes, mRNA and protein expressions of voltage-gated potassium channels (Kv1.5 channel), were measured. The concentrations of monocyte chemo-attractant protein-1 (MCP-1) and transforming growth factor-beta1 (TGF-β1) were detected. Furthermore, pulmonary arterial smooth muscle cells (PASMCs) were isolated and cultured with or without hypoxia pretreated with or without 5-HD or/and Kv1.5 inhibitor 4-aminopyridine (4-AP). Mitochondrial membrane potential (Δψm) and the proliferation of PASMCs were detected.

**Results:**

5-HD significantly prevented the development of PAH by blocking the mitochondrial membrane depolarization, increased the expression of voltage-gated potassium channels, and reduced pulmonary hypertension mediated by TGF-β1 or MCP-1 signaling pathway.

**Conclusion:**

The MitoK_ATP_ plays an important role in the development of PAH and may be therapeutic target for the treatment of disease.

## Background

PAH is a severe form of lung disease, leading to right ventricular failure or death. Hypoxic pulmonary vasoconstriction is the very important physiological phenomenon which optimizes ventilation–perfusion matching. However, sustained pulmonary vasoconstriction and pulmonary artery remodeling could lead to PAH, as a complex phenomenon involving multiplicity of interacting mechanisms which has still not been elucidated. There is growing evidence that mitochondria as a key event is responsible for initiation of PAH [1-4]. Mitochondrion as the cell energy factory is sensitive to hypoxia and plays a crucial role in hypoxia-mediated cell proliferation and apoptosis [5]. The Kv channel of PASMCs can directly respond to the oxygen tension by controlling resting membrane potential and regulating vasomotor. The Kv1.5 was found as the primary oxygen-sensitive subtype [6], probably involved in hypoxic PAH. The expression of TGF-β1 in lung tissues regulated by hypoxia could modulate PASMCs proliferation, which is tissue-specific and modulated by the cellular micro-environment [7]. Reactive oxygen species (ROS) could be detected in both the mitochondrial matrix and cytosol, which augment TGF-β-induced profibrotic gene expression [8]. MCP-1 is a potent chemo-attractant and an activator for mononuclear cells. Mitochondrial uncoupling under hypoxia-independent adipocytes can reduce MCP-1 release through induction of endogenous ER stress and activation of AMP-activated potein kinase [9]. TGF-β and MCP-1 were found to be closely associated mitochondrion and with the formation of PAH [10].

The mitoK_ATP_ is a key factor in the control of mitochondrial membrane potential (∆ψm), which is sensitive to hypoxia. The previous study showed that hypoxia could activate mitoK_ATP_ channels cause the depolarization of ∆ψm and block the mitochondrial electron transport chain, stimulate mitochondrial H_2_O_2_ overproduction, increase cytochrome C accumulation, or increase the expression of HIF-1 α [11,12], resulting in an imbalance between proliferation and apoptosis in hPASMCs. It has been reported that opening or closure of mitoK_ATP_ may be involved in the modulation of mitochondrial protein complexes such as ROS-producing complex II and ANT-regulated mitochondrial permeability transition pore [13]. Administration of 5-HD could inhibit mitoK_ATP_ and prevent from hypoxia- and diazoxide-induced proliferation through voltage-gated K + channel [14,15]. So far, there has not been any reports as to whether mitoK_ATP_ plays any important role in vivo, and the studies about the modulation mechanism of mitoK_ATP_ on TGF-β and MCP-1 are rarely documented. The mechanism by which TGF-β1 and MCP-1 increases during hypoxia is unclear. The present study aims at investigating the important role of mitoK_ATP_ in hypoxic-induced PAH in vivo, to measure alterations of Kv1.5 expression, TGF-β1 and MCP-1.

## Methods

### Reagents

5-HD with the purity of 99%, 4-aminopyridine (4-AP), and rhodamine-123 (R-123) were purchased from Sigma (USA). Polyclonal mouse anti-rat α-actin antibody (α-SM actin), polyclonal goat anti-rat Kv1.5 antibody, goat anti-rat MCP-1 antibody, and rabbit anti-rat TGF-β1 antibody were purchased from Santa Cruz (USA). Cell counting kit-8 (CCK) was purchased from Tongue Chemical Institutes (Japan).

### Animal model

Thirty adult male Sprague–Dawley rats, weighing 250–300 grams, were obtained from Hays Lake Animal Co. Ltd (Shanghai, China). Rats in the present study were handled in accordance with the guidelines of Wenzhou Medical College and National Institutes of Health Guide for the Care and Use of Laboratory Animals. The study was approved by the Animal Ethics Committee of Wenzhou Medical College including permit number SCXK (Zhejiang 2005–0019 150). Twenty four rats were randomly divided into three groups (n = 8 per group): 1) animals with manipulations with normoxia (Controls); 2) animals with hypoxia and pretreated with vehicle (Hypoxia + V); or 3) animals with hypoxia and pretreated with 5-HD (Hypoxia +5-HD). Animals were exposed to normoxia or hypoxia for one week and then intraperitoneally injected (i.p.) with vehicle (0.9% saline) or 5-HD (5 mg/kg/day) with a continuous hypoxic exposure for 3 weeks. Hypoxia was achieved by placing animals in a hypoxic chamber with the oxygen level at 10 ± 0.5%. PASMCs were isolated and cultured from six rats randomly with normoxia, hypoxia pretreated with vehicle, or hypoxia pretreated with 5-HD at 500 mmol/L, or hypoxia with 5-HD and 4-AP (Kv1.5 inhibitor) at 10 mmol/L for 24 hours.

### Measurements of pulmonary hypertension

The animals were weighed and intra-peritoneally anesthetized with chloral hydrate at the dose of 1 g/kg. The right jugular vein was dissected and cannulated with the heparinized polyethylene catheter-1106 which was connecting with YL-4-type pressure transducer linked to the Power lab data acquisition unit (AD Instruments, Australia). The waveforms of the pulmonary arterial pressure were recorded to calculate the mean pulmonary artery pressure (mPAP), after the waveform was stable [16]. Levels of mPAP were measured and the left lung tissue was then harvested and fixed in 4% paraformaldehyde, dehydrated, embedded in paraffin, sliced and stained with anti-α-SM-actin monoclonal antibody (Santa Cruz, USA, 1:150) to detect the cytoskeleton protein α-actin expression of PASMCs. The secondary antibody in the absence of the primary antibody was used as a negative control. Ten alveolar ducts (ADs) with a diameter of 20- 150 μm and positive expression of brown cytoplasm were randomly selected to determine the average optical density (A) of the α-SM-actin protein expression using Image-ProPlus 6.0 software. A hilar-level cross-section of the lung tissue was cut into a size of 1 × 1 × 2 mm, which was immobilized with 2.5% glutaraldehyde and postfixed with 1% osmium tetroxide, and then embedded with Epon812. Sliced tissues were processed with LKV-V-type ultra-thin slicing machine, sliced and dyed. The ultra-structure of pulmonary arterioles was examined with H-600 transmission electron microscope.

### Measurement of MCP-1

The serum levels of MCP-1 were measured by enzyme linked immunosorbent assay (ELISA) at the optical density (absorbance, A) of 450 nm wavelength readout, and expressed by pg/ml, according to the instructions of ELISA.

### Measurement of TGF-β1

Immuno-histochemisty was performed according to the manufacturers recommendations (Santa Cruz Biotechnology, USA) to detect the expression of TGF-β1 in pulmonary artery. The diluted concentration of antibody TGF-β1 was 1:100, and the secondary antibody in the absence of the primary antibody was used as the negative control. Small pulmonary arteries with diameters of 20 -150 μm were analyzed with Image-Pro Plus 6.0 software. 6–8 fields of vision were selected from each section for quantitative analyses.

### Reverse transcription-polymerase chain reaction

Total RNA was rapidly obtained from pulmonary arteries using Trizol (Invitrogen Life Technologies, USA). RNA concentration was quantified using absorbance at 260 nm and the ratio of the absorbance at 260 and 280 nm (A260/280) was used to assess the purity of RNA. Reverse transcription was performed at 42°C for 1 h and then at 95°C for 5 min. The resulting cDNA was subjected to PCR amplification with specific primers. PCR was performed with the M7122 Real-Time PCR instrument (Promega Life Technology, USA). The primers used for Kv1.5 were as follows: 5′-CCA GCG AGT CCT CAT AAA CA-3′ for the forward primer, and 5′-CAC TAT GGC GAT GGC TCTT-3′ for the reverse primer. The predicted amplification product size of Kv1.5 was 409 bp, and the product size of β-actin was 457 bp. The following cycle parameters were used: 5 min at 94°C, followed by 40 cycles of 1 min at 94°C and 45 s at 54°C and 72°C for 45 s. Amplified products were visualized by 1.5% agarosgel electrophoresis with ethidium bromide staining. The intensity of PCR product bands was quantified by scanning densitometry. The relative expression level of Kv1.5 mRNA was normalized to that of β-actin. The statistical analysis of the PCR result was analyzed using the Gelpro32 Software.

### Western blot

Pulmonary arteries were homogenized and then lysed using a strong Radio Immunoprecipitation Assay lysis buffer and Phenylmethanesulfonyl fluoride. After centrifuged at 12,000 rpm for 20 min at 4°C, the supernatant was discarded, protein were quantified by the method of BCA protein assay kit (Biyuntian biotechnology company, Jiangsu, China) according to the manufacturer’s instructions. An equal amount of proteins (75 μg) were separated by 10% SDS–polyacrylamide gel electrophoresis and transferred onto polyvinylidene fluoride membrane (Millipore, MA), and blocked with triethanolamine-buffered saline containing 5% fat-free milk powder for 1 hour. The membrane was then incubated with specific primary antibodies: goat anti-Kv1.5 polyclonal antibody at 1:200 overnight at 4°C, and then the corresponding secondary antibody (HRP-conjugated rabbit anti-goat IgG 1:5000 (Beijing Zhongshan Biotechnology Co., Beijing, China) was added and incubated for 2 h, The membrane was washed with tris-buffered saline tween buffer for 10 min three times. The immuno-reactive band was detected with BeyoECL Plus reagents. Enhanced chemiluminescence films were processed using Gelpro32 image analysis system.

### Cultured cells

The animals were anesthetized with chloral hydrate. Pulmonary arteries at diameters of 2–3 mm were isolated from the surrounding parenchyma and smooth muscle bundles were dissected from the artery. Cells were digested with 0.2% collagenase and subcultured. The dissociated cells were resuspended in DMEM containing 20% FBS on culture flasks and maintained at 37°C in 5% CO_2_ and at 95% humidity. The culture medium was changed every 2 to 3 days until smooth muscle cells were confirmed using immuno-fluorescence staining with mouse anti-a-actin polyclonal antibody. Cells were exposed to normoxia, hypoxia, hypoxia in the presence of 5-HD at 500 mmol/L, and hypoxia in the presence of 5-HD with 4-AP at 10 mmol/L for 24 hours. Normoxia was achieved by using a gas mixture of 74% N2, 5% CO_2_, and 21% O_2_, and hypoxia was obtained by using a gas mixture of 93% N2, 5% CO_2_, and 2% O_2_.

### Measurement of PASMC proliferation

Cell proliferation was measured by CCK-8 assay (Dojindo, Japan)based on the colorimetric measurement of formazan dye formed from WST-8. Cells were seeded into a flat-bottomed 96-well plate at a density of approximately 1 × 10^6^ cells per millilitre, and incubated for 24 h in serum-free medium. Cells were exposed to normoxia or hypoxia in the presence of 5-HD or/and 4-AP for 24 h. Cells were incubated with CCK-8 cell counting kit for 90 min in the absence of light at the end of treatments. The absorbance of the solubilized product at 450 nm was measured using the ELISA reader (Bio-TEK, USA). All the measurements were conducted at least three times.

### Mitochondrial membrane potential

The potential of mitochondrial membrane (Δψm) was measured as described previously [17]. Cells were exposed to 5-HD or 5-HD plus 4-AP under hypoxia for 24 hours and then incubated with rhodamine123 at 10 mg/ml (R-123; Sigma, USA) for 30 min at 37°C. R-123 fluorescence was excited at 488 nm and measured at 530 nm with a confocal microscope (Olympus, Japan). The intensity of R-123 fluorescence was taken as an indicator of inner Δψm. The increase in R-123 fluorescence represents the depolarization of Δψm.

### Data analysis

Statistical analyses were performed by SPSS software. All data are expressed as mean ± SD. P values less than 0.05 was considered as statistically significant. One-way analysis of variance (ANOVA) was used for the comparisons of variance among several groups. Dummett’s test was used for multiple comparisons.

## Results

**Figure 1:**
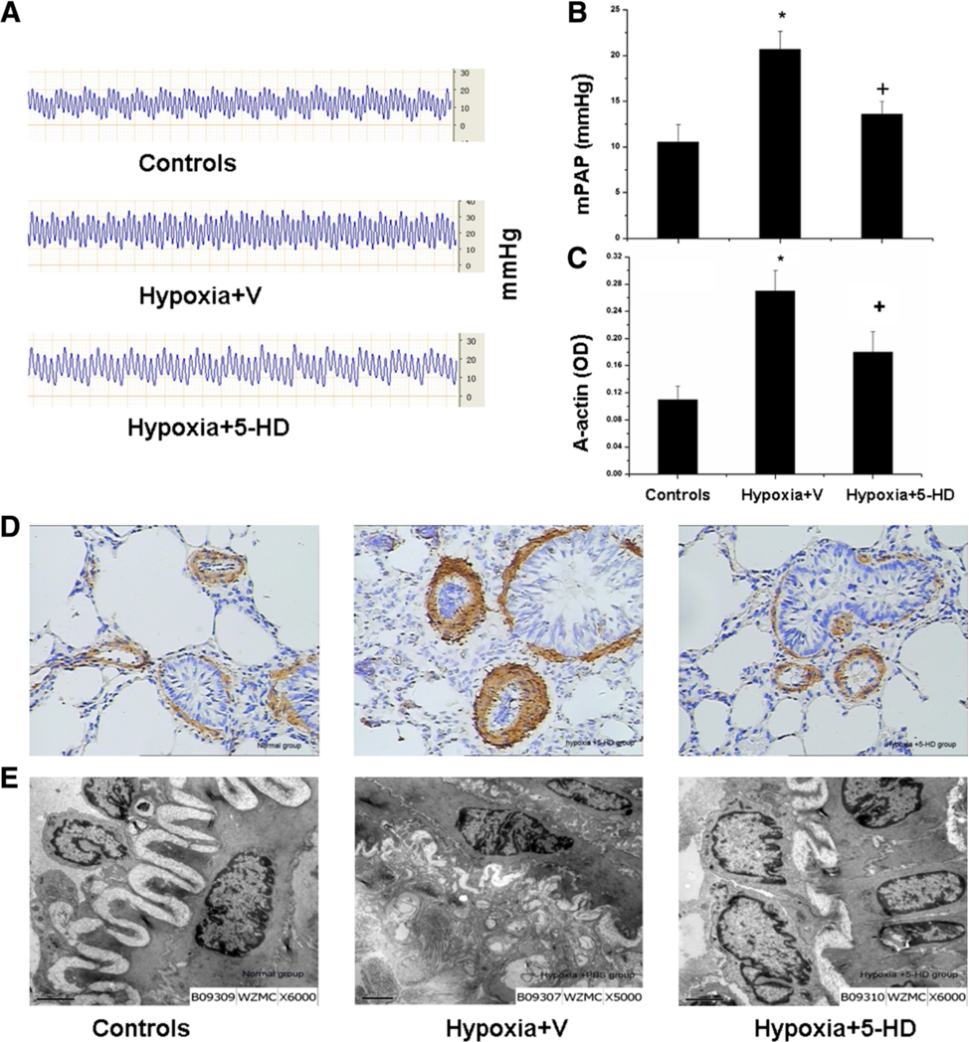
**Effects of mitochondrial ATP-sensitive potassium channel inhibitor (5-HD) on the development of pulmonary hypertension. (A)** Pulmonary arterial pressure (PAP) according to a right cardiac catheterization procedure; **(B)** The mean values of PAP (mPAP); **(C)** The Optimal density (OD) of a-actin; **(D)** Immuno-histochemical staining of smooth muscle a-actin expression (×200); **(E)** Ultrastructural morphology in rat lung tissues (×6000). Rats were treated with vehicle and normoxia as Controls, with vehicle and hypoxia (Hypoxia + V), or with 5-HD and hypoxia (Hypoxia + 5-HD). Each group includes 8 animals and values were expressed as mean ± SD. *and + stand for p values less than 0.05, as compared with Controls and Hypoxia + V group, respectively.

**Figure 2:**
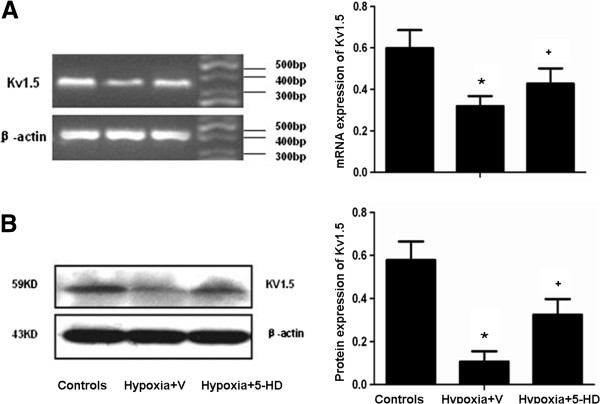
**Effect of 5-HD on mRNA (A) and protein expression (B) of Kv1.5 in pulmonary arteries.** Rats were treated with vehicle and normoxia as Controls, with vehicle and hypoxia (Hypoxia + V), or with 5-HD and hypoxia (Hypoxia + 5-HD). Each group includes 8 animals and values were expressed as mean ± SD. *and + stand for p values less than 0.05, as compared with Controls and Hypoxia + V group, respectively.

**Figure 3:**
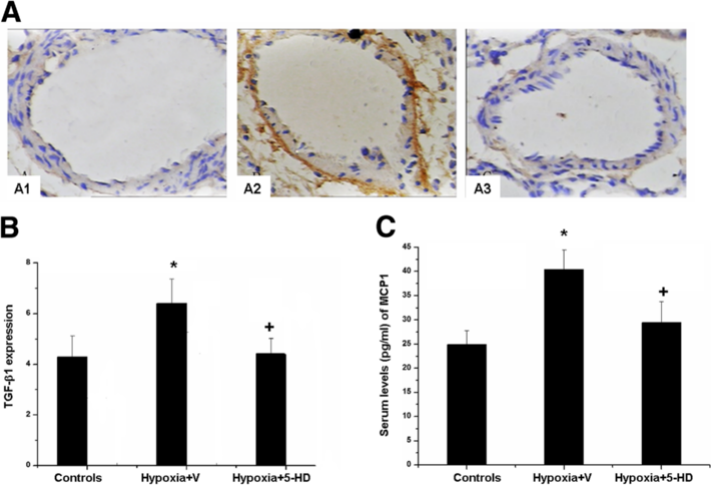
**Effect of 5-HD on the localization of TGF-β1 and MCP-1 protein expression. (A)** Positive expression of TGF-β1 was mainly detected in pulmonary arteries and significantly increased in medium and small pulmonary arteries in Hypoxia + V group. **(B)** Treatment with 5-HD could prevent from hypoxia-induced expression of TGF-β1 in the pulmonary artery. **(C)** Serum levels of MCP-1 in Hypoxia + V group significantly increased vs Controls, while not in Hypoxia + 5-HD group. Each group includes 8 animals and values were expressed as mean ± SD. *and + stand for p values less than 0.05, as compared with Controls and Hypoxia + V group, respectively.

Levels of mPAP in Hypoxia + V group significantly increased as compared to Controls (p <0.05), Treatment of the mitoK_ATP_ channel inhibitor 5-HD at 5 mg/kg/day for 3 weeks significantly prevented increased mPAP in hypoxic rats (p <0.05; Figure 1A and B). Histo-pathological examinations of lung tissues revealed that walls of distal pulmonary arteries with positive expression of brown cytoplasm became significantly thickened in Hypoxia + V group, as compared with Controls, which was significantly inhibited by the treatment with 5-HD. The expression of smooth muscle-specific α-actin in pulmonary arterioles significantly increased in Hypoxia + V group, but not in group treated with 5-HD (p <0.05; Figure 1C and 1D). Flat endothelial cells, regular intimal elastic lumina, small PASMCs, and a few collagen fibers between PASMCs were detected in lung tissues from Controls. Swollen endothelial cells with a cubic shape, an amount of vacuolization along with the basal layer, or the crooked lumina were observed in Hypoxia + V group. Hypoxia-induced changes were significantly alleviated by treatment with 5-HD (Figure 1E).The expression of Kv1.5 mRNA in pulmonary arteries of Hypoxia + V group significantly reduced as compared with Controls (p < 0.05; Figure 2A), while not in group with 5-HD. Expression of Kv1.5 protein significantly decreased in Hypoxia + V group, while the treatment with 5-HD could significantly inhibit hypoxia-induced low protein expression, as compared with Hypoxia + V (Figure 2B).Positive expression of TGF-β1 was mainly detected in pulmonary arteries and significantly increased in medium and small pulmonary arteries in Hypoxia + V group (p <0.05 vs controls; Figure 3A and B). Treatment with 5-HD could prevent from hypoxia-induced expression of TGF-β1 in the pulmonary artery (p <0.05 vs Hypoxia + V). Serum levels of MCP-1 in Hypoxia + V group significantly increased vs Controls (p <0.05), while not in Hypoxia + 5-HD group, as show in Figure 3C.

**Figure 4:**
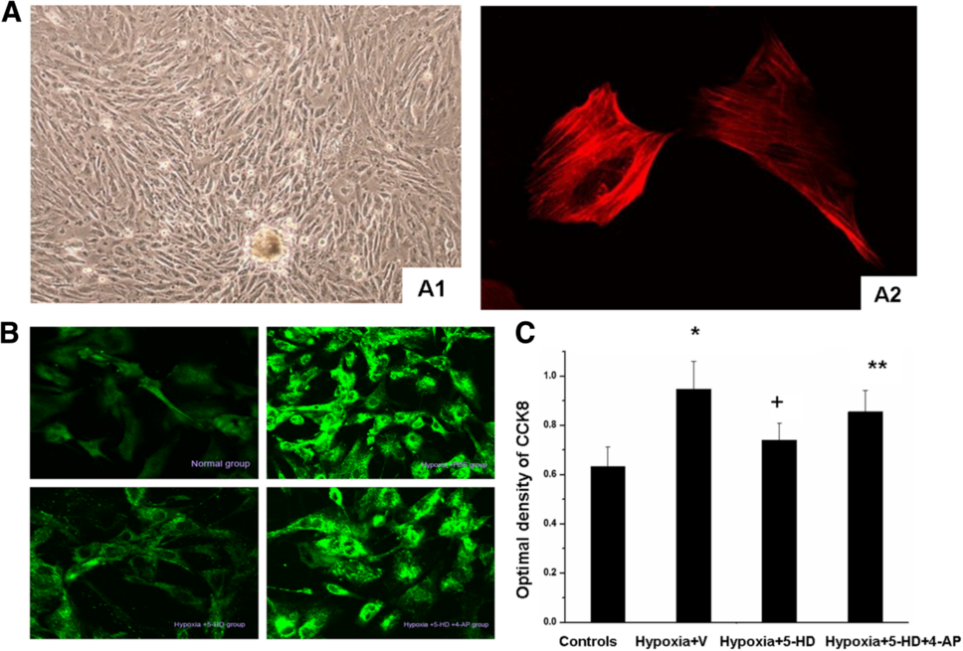
**Effect of 5-HD on Δ ψ m and proliferation in PASMCs. (A)** Morphology of isolated PASMCs. (A1) PASMCs appeared spindle- shaped, the typical “hill-and-valley” (phase contrast microscopy × 100); (A2) Positive expression of PASMCs α-actin appearance (Laser Confocal Microscope × 400). **(B)** Effect of mitochondrial ATP-sensitive potassium channel inhibitor (5-HD) on hypoxic depolarization of mitochondrial membrane potential in PASMCs treated with vehicle and normoxia as Controls, with vehicle and hypoxia (Hypoxia + V), with 5-HD and hypoxia (Hypoxia + 5-HD), or with the combination of 5-HD and Kv1.5 inhibitor (4-AP) and hypoxia (Hypoxia + 5-HD + 4-AP), measured by the intensity of R-123 fluorescence using a laser-scanning confocal microscope (B; ×200). **(C)** The proliferation of PASMC was measured by CCK-8 colorimetric assay. *and **stand for p values less than 0.05 and 0.01, as compared with Controls, respectively, and + stands for p values less than Hypoxia + V group.

Cultured PASMCs showed spindle-shaped features, as well as the characteristic “hill and valley” appearance with a prominent central oval nucleus under inverted phase contrast microscope and positive staining of smooth muscle-specific α-actin in Figure 4A. R-123 fluorescence intensity increased in PASMCs 24 hours after hypoxia, as an indication of depolarization of Δψm, which was significantly inhibited by 5-HD treatment. The R-123 fluorescence intensity increased in the concomitant stimulation of 4-AP and 5-HD, as compared with Controls or 5-HD alone (Figure 4B). 5-HD attenuated hypoxia-induced increase in R-123 fluorescence intensity. These results indicated that hypoxia opens mitoK_ATP_ and induces an increase in ∆ψm in PASMC, while 5-HD reverses the depolarization of Δψm by voltage-gated potassium channels (Kv1.5 channel). PASMC proliferation measured by CCK-8 colorimetric assay significantly increased in Hypoxia + V and Hypoxia + 5-HD + 4-AP groups, but not in Hypoxia + 5-HD alone, as compared with Controls (p <0.05, Figure 4C).

## Discussion

MitoK_ATP_ channels play an important role in the control of membrane potential, energy production, homeostasis and ROS generation [18]. It has been suggested that the activation of the mitoK_ATP_ channel is regulated with the decline of intracellular ATP levels and increase of intracel-lular levels of nucleoside diphosphates in cardio-myocytes [19]. The opening of mitoK_ATP_ could protect different cell types against apoptosis, including cardiac myocytes, renal epithelial cells, cere-bellar granule neurons, or skin cells [20-23]. Hypoxia might provoke proliferation and lessen apoptosis through mitochondrial apoptotic or proliferative signaling pathway in hPASMCs [24]. 5-HD as a specific mitoK_ATP_ inhibitor could inhibit PASMC mitochondrial ATP sensitive potassium channel and the depolarization induced by hypoxia [14]. PAH is a severe disease and accounts for most deaths of chronic obstructive pulmonary diseases, and remodeling of the pulmonary vessel wall contribute to the increased pulmonary vascular resistance. In the present study, we successfully established rat model of PAH. Values of mPAP significantly increased compared with controls. Results showed that pretreatment of rats with 5-HD could block hypoxia-induced increase of mPAP, the expression of smooth muscle-specific a-actin in pulmonary arteries, and alterations of PASMC ultrastructure. The present study in vitro showed that hypoxia induced depolarization of Δψm and promote PASMCs proliferation, which was significantly inhibited by 5-HD treatment. In the current study, Changes in Δψm, as indicated by the intensity of R-123 fluorescence. It indicated that mitoK_ATP_ channels was involved in and/or responsible for hypoxia-induced pulmonary vascular remodeling and the development of PAH. The inhibition of mitoK_ATP_ channel with 5-HD could block hypoxic-cellular responses in PASMCs and prevent the development of PAH.

MitoK_ATP_ channels through ROS indirectly inhibit the Kv1.5, open the voltage-gated calcium channels and increase intracellular calcium influx, and caused PASMC contraction and proliferation [25]. Down-regulation of Kv1.5 channels was noticed to contribute to the development PAH by promoting PA vasoconstriction and remodeling [26], and 5-HD could increase the expression of Kv1.5 channel in hPASMCs [15]. The present study in vivo demonstrated that the mitoK_ATP_ channel inhibitor 5-HD could prevent the development of hypoxia-induced PAH by the up-regulation of the mRNA and protein expression of Kv1.5 in animals with hypoxia-induced. To further study the mechanism of 5-HD, we investigated Kv1.5 channel expression induced by hypoxia and effects of 4-AP(Kv1.5 inhibitor)in cultured PASMCs. The results further more evidenced that the combination of 4-AP and 5-HD increased depolarization of Δψm and increased proliferation, which mean that 5-HD could reverse the depolarization of Δψm by Kv1.5 channel.

Excessive TGF-beta signaling can initiates profibrotic gene expression. The hypoxia-induced over-expression of TGF-β1 was proposed to increase the expression of NOx4 and oxygen free radicals through Smad2/3 signal pathways and regulate the proliferation of PASMCs, responsible for pulmonary artery reconstruction [27,28]. The results of the current study showed that the production of TGF-β1 from pulmonary arteries increased in hypoxia, which can be prevented by 5-HD. It seems 5-HD could influence TGF-β1-associated signal pathway in pulmonary arteries.

MCP-1 may play a role in the initiation and/or progression of PAH. We found that hypoxia could increase serum levels of MCP-1, which may be involved in the interaction between leukocytes and pulmonary endothelia to changes of pulmonary vascular smooth muscle cells, e.g. increased vascular wall thickness, distal non-muscular artery to muscularization, or pulmonary vascular to stenosis, especially for the small pulmonary arteries [29,30]. Induction of mitochondrial reduces MCP-1 release in mature 3 T3-L1 adipocytes maybe through AMPK-related pathways [9]. The results of the present study confirmed that 5-HD could prevent hypoxia-induced elevated serum levels of MCP-1, accompanied by reduction of pulmonary vascular remodeling. It has been proposed that the circulating levels of MCP-1 was regulated by MitoK_ATP_, although the exact mechanism which 5-HD altered systemic levels of those cytokines induced by hypoxia remains unclear.

## Conclusion

**Figure 5:**
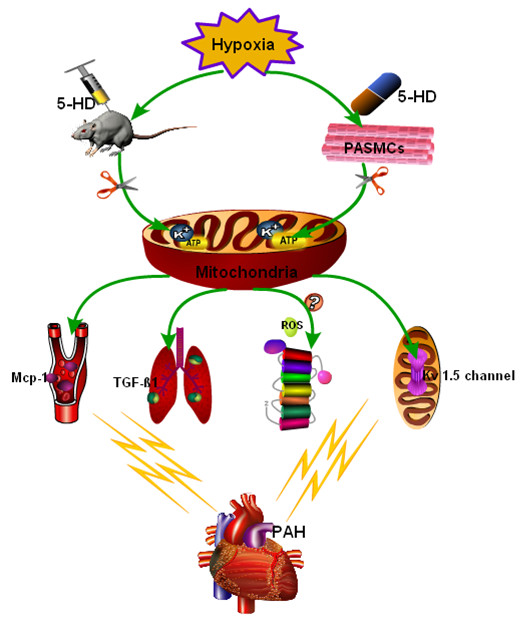
**5-HD down-regulates hypoxic-induced pulmonary artery hypertension.** 5-HD could inhibit mitoK_ATP_ channels and block the hypoxic depolarization of mitochondrial membrane potential of PASMCs via Kv1.5 channels, inhibiting the circulating inflammatory cytokines MCP-1 and down-regulating TGF-β1 signaling way, or inhibiting PAH by oxygen free radicals (ROS)-related signal pathways.

In conclusions, we provided an in vivo evidence that the inhibition of the mitoK_ATP_ channel could prevent hypoxia-induced depolarization of mitochondrial membrane potential via Kv1.5 channels, circulating of MCP-1, and regulation of TGF-β1 signaling way. Other factors (such as MPTP, PKC-a, ROS, HIF-1 α, NOx4, Smad2/3) might be also involved in the mechanism of hypoxic pulmonary vascular remodeling. The treatment of the mitoK_ATP_ channel inhibitor could also alter the hypoxia-induced PASMC proliferation, pulmonary vascular remodeling, and the development of PAH, as explained in Figure 5. Thus, mitoK_ATP_ channels may play an important role in the formation of hypertension and should be considered as one of therapeutic targets for PAH.

## Abbreviations

4-AP: 4-aminopyridine; 5-HD: 5-Hydroxydecanoate; CCK-8: Cell counting kit-8; ELISA: Enzyme linked immunosorbent assay; Kv: Voltage-gated potassium channels; MCP-1: Monocyte chemoattractant protein-1; mitoK_ATP_: Mitochondrial ATP-sensitive potassium channel; mPAP: Mean pulmonary arterial pressure; PAH: Pulmonary artery hypertension; PASMCs: Pulmonary artery smooth muscle cells; R-123: Rhodamine-123; ROS: Reactive oxygen species; TGF-β1: Transforming growth factor-beta 1; Δψm: Mitochondrial membrane potential.

## Competing interests

The authors declare that they have no competing of interests.

## Authors’ contributions

Conceived and designed the study: YL, HLH and Chengshui C; Performed the biological experiments: LD; BW; Animal model: JC; Chaolei C; Statistical analysis: HZ, YL, QL, LZ. Wrote the paper: LD and LS. All authors read and proofed the final manuscript.
